# A randomized controlled trial on the efficacy of carbohydrate-reduced or fat-reduced diets in patients attending a telemedically guided weight loss program

**DOI:** 10.1186/1475-2840-8-36

**Published:** 2009-07-18

**Authors:** Sabine Frisch, Armin Zittermann, Heiner K Berthold, Christian Götting, Joachim Kuhn, Knut Kleesiek, Peter Stehle, Heinrich Körtke

**Affiliations:** 1 Klinik für Thorax- und Kardiovaskularchirurgie, Herzzentrum NRW, Ruhr Universität Bochum, Bad Oeynhausen, Germany; 2 Charité-Universitätsmedizin Berlin; Interdisziplinäres Stoffwechsel-Centrum; Lipidambulanz, Lipidapherese und Ernährungsmedizin, Berlin, Germany; 3 Institut für Laboratoriums- und Transfusionsmedizin, Herzzentrum NRW, Ruhr Universität Bochum, Bad Oeynhausen, Germany; 4 Institut für Ernährungs- und Lebensmittelwissenschaften, Universität Bonn, Germany

## Abstract

**Background:**

We investigated whether macronutrient composition of energy-restricted diets influences the efficacy of a telemedically guided weight loss program.

**Methods:**

Two hundred overweight subjects were randomly assigned to a conventional low-fat diet and a low-carbohydrate diet group (target carbohydrate content: >55% energy and <40% energy, respectively). Both groups attended a weekly nutrition education program and dietary counselling by telephone, and had to transfer actual body weight data to our clinic weekly with added Bluetooth^® ^technology by mobile phone. Various fatness and fat distribution parameters, energy and macronutrient intake, and various biochemical risk markers were measured at baseline and after 6, and 12 months.

**Results:**

In both groups, energy intake decreased by 400 kcal/d compared to baseline values within the first 6 months and slightly increased again within the second 6 months. Macronutrient composition differed significantly between the groups from the beginning to month 12. At study termination, weight loss was 5.8 kg (SD: 6.1 kg) in the low-carbohydrate group and 4.3 kg (SD: 5.1 kg) in the low-fat group (p = 0.065). In the low-carbohydrate group, triglyceride and HDL-cholesterol levels were lower at month 6 and waist circumference and systolic blood pressure were lower at month 12 compared with the low-fat group (P = 0.005–0.037). Other risk markers improved to a similar extent in both groups.

**Conclusion:**

Despite favourable effects of both diets on weight loss, the carbohydrate-reduced diet was more beneficial with respect to cardiovascular risk factors compared to the fat-reduced diet. Nevertheless, compliance with a weight loss program appears to be even a more important factor for success in prevention and treatment of obesity than the composition of the diet.

**Trial registration:**

Clinicaltrials.gov as NCT00868387

## Background

The burden of overweight (body mass index (BMI) ≥ 25 kg/m^2^) and obesity (BMI ≥ 30 kg/m^2^) has dramatically increased during the last decades [1]. The WHO estimated that in 2006 worldwide 1.6 billion adults were overweight and 400 million adults were obese [2]. In the United States, two in three adults have a BMI ≥ 25 kg/m^2 ^[3]. This situation is primarily due to an excess energy intake, low energy expenditure, or both. It has recently been assumed that high carbohydrate intake, particularly refined carbohydrates, increases the risk of obesity, type 2 diabetes mellitus, and metabolic syndrome by exaggerating postprandial glycemia [4-6].

For several decades, medical societies and governmental health societies had recommended energy-restricted diets high in unrefined carbohydrates and fibre to prevent or treat obesity [7,8]. Based on the aforementioned new findings, these official recommendations have now been questioned [9-11]. Several randomized, controlled studies have shown greater weight loss and better improvement of cardiovascular risk parameters with energy-restricted low-carbohydrate diets compared to energy-restricted low-fat diets after treatment periods of six to twelve months [12-15].

Beside macronutrient relations, efficacy of weight loss programs depends on care and control [16]. At present, the weight watchers program is the only evidenced-based strategy to treat obesity. Weight loss of 4.3 kg in the participants of this program compared to weight loss of only 1.3 kg in the control group has been reported over a period of twelve months [17]. The SMART (**S**chlank **M**it **A**ngewandte**r T**elemedizin)-study is based on a telemedically guided weight loss program that has been established at our clinic [18]. Here, we present those results from the SMART-study, where we have investigated whether or not a carbohydrate-restricted telemedically guided weight loss program results in a more pronounced weight loss and influences metabolic risk markers more beneficial than a fat-restricted diet does.

## Methods

### Participants

**Figure 1:**
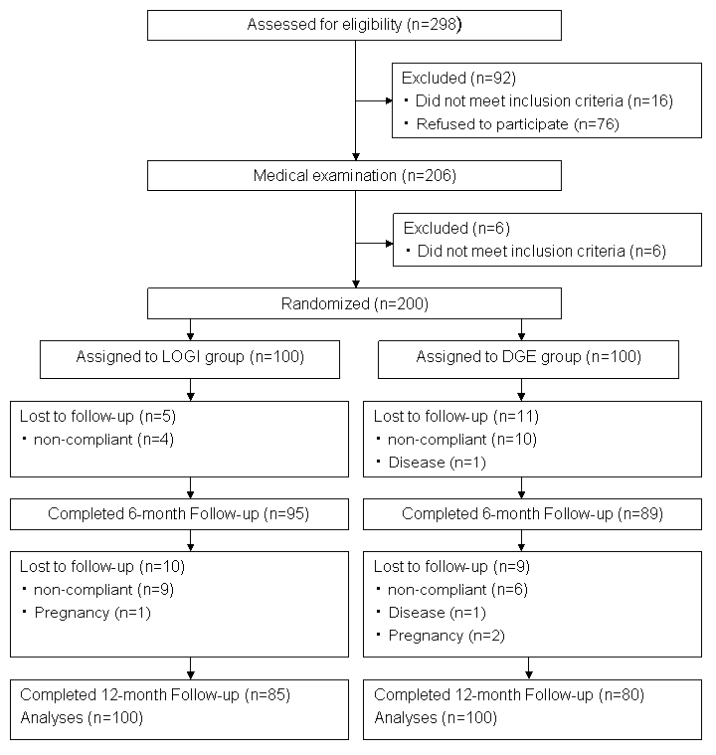
**Participant flow through the trial**.

This study was conducted between December 2005 and November 2006 at the Heart Center North Rhine-Westphalia, Institute for Applied Telemedicine, Bad Oeynhausen, Germany. Recruitment began in November 2005 by advertisements in local newspapers and by providing information sheets at different local health insurance offices. The criteria for eligibility were an age of 18 to 70 years and a BMI (calculated as weight in kilograms divided by height in meters squared) > 27 kg/m^2^. We excluded patients with a history of any cardiovascular symptomatology. We also performed an exercise electrocardiogram for exclusion of ischemia, and a stress echocardiography when appropriate. Moreover, we excluded patients with cholelithiasis, urolithiasis, insulin dependent diabetes mellitus, and pacemaker implantation. In addition, pregnancy, lactation, and vegetarianism were exclusion criteria. Further exclusion criteria were the participation in another weight loss program and medical treatment for weight reduction. Of a total number of 298 persons who were initially interested in attending the study 76 persons refused to participate after a first phone screening. Additional 16 persons did not meet the inclusion criteria. For the same reason six other persons were excluded at the baseline investigation at our clinic. In total, 200 persons were thus included in our study (Figure 1). All participants gave written informed consent to the study procedures, which were approved by the Ethics Committee of the Ruhr-University Bochum, Faculty of Medicine, Germany.

### Study design

Participants (n = 200) were randomly assigned by computer-generated random number lists in two equal groups: the LOGI group was assigned to a low-carbohydrate diet developed by Ludwig et al. [9] and modified by Worm [19]; the DGE group received a conventional low-fat diet according to recommendations issued by the *Deutsche Gesellschaft für Ernährung *[20]. The target macronutrient composition in the LOGI group was less than 40% of total energy intake (% energy) from carbohydrates, more than 35% energy from fat, and 25% energy from protein [21]. The target macronutrient composition in the DGE group was more than 55% energy from carbohydrates, less than 30% energy from fat, and 15% energy from protein. We instructed all participants in an ambulatory training session and delivered diet books to all participants about their respective diets [19,22,23]. All participants were advised to reduce their daily energy intake by at least 500 kcal. At study entry, each participant received an electronic scale (TC-100, I.E.M., Stolberg, Germany, mean deviation ± 0.2 kg with standard deviation of 0.06 kg) with added Bluetooth^® ^technology. Weekly, the actual body weight data had to be sent to the Institute of Applied Telemedicine using a mobile phone. The weight reduction program consisted of weekly nutrition education and dietary counselling by phone with a nutritionist during the first six months. These calls were standardized according to weekly items about the respective diet, eating habits, and weight reduction, which allowed individual and problem-oriented advice. Each participant arranged her/his own appointment for weighting and calling. This regular weekly support was stopped during the second half-year. The study subjects were responsible for buying and preparing foods by themselves. At baseline, after six months, and after twelve months, each subject attended an out-patient medical visit where body weight and height were measured in underwear on a calibrated electronic clinical scale (model 920, SECA GmbH & co. kg., Hamburg, Germany; tolerance ± 0.2 kg). Moreover, waist circumference was assessed by standard procedures using a 150-cm anthropometric measuring tape, and body composition by bioelectrical impedance analysis (Multifrequency Analyzer Nutrigard M, Data Input GmbH, Darmstadt, Germany). Blood pressure was manually determined using a cuff and stethoscope. Blood samples were drawn from the antecubital vein after a 12-h overnight fast. After centrifugation at room temperature for 20 minutes (1500 × g), aliquots of serum and EDTA-plasma samples were frozen consecutively and stored at -80°C until analyzed. We defined the presence of a metabolic syndrome after determination of waist circumference, blood levels of triglycerides, high density lipoprotein (HDL)-cholesterol, and fasting plasma glucose, blood pressure, and respective medical treatment according to current guidelines [24]. Medical history and medical treatment were assessed using hospital documents.

At baseline and after 1, 3, 6, and 12 months, we assessed energy and nutrient intake (diet compliance) by the use of a 3-day validated food record [25] and the amount of daily physical activity by using a standardized, validated questionnaire [26]. We calculated metabolic rate by multiplying the time of exercise with their respective metabolic equivalent (MET) using adequate schedules to determine METs [27,28].

We considered weight loss and loss of fat mass as primary endpoints. Metabolic and cardiovascular risk markers such as waist circumference, blood pressure, blood lipids, and parameters of glucose metabolism were considered secondary endpoints.

### Biochemical Analyses

We measured serum levels of total cholesterol, high-density lipoprotein (HDL)-cholesterol, low density lipoprotein (LDL)-cholesterol, triglycerides, glucose, and fructosamine using the Architect autoanalyzer (Abbott, Wiesbaden, Germany), and glycated hemoglobin (HbA1c) using the autoanalyzer HA-8160 (Menarini Diagnostics, Berlin, Germany). We used enzyme-linked immuno assay kits to assess proinsulin (IBL, Hamburg, Germany) and adiponectin (R&D Systems GmbH, Wiesbaden-Nordenstadt, Germany). All intra- and interassay coefficients of variation were below 10.0%.

### Statistical Analysis

We expressed categorical variables as percentage rates and continuous variables as mean ± standard deviation or standard error of the mean, when appropriate. Because several variables such as fat-free mass, diastolic and systolic blood pressure, glucose, HbA1c, proinsulin, triglycerides, HDL-cholesterol, and adiponectin were not normally distributed as tested by the Kolmogorov-Smirnov test, these data were normalized using logarithmic transformation. We used the unpaired t-test to compare continuous values of the study groups at baseline. For comparative evaluations of categorical variables, we used the Chi Square test. Results of anthropometric, clinical, and biochemical parameters at the 6-month and 12-month examinations are presented as change from baseline. The unpaired t-test and the paired t-test were used to specify those time points with significant differences between groups or within groups, when appropriate. A 2-factor analysis of covariance was used to compare anthropometric, clinical, and biochemical parameters between the LOGI and DGE groups with its respective baseline value and with fat-free mass and sex distribution as covariates. Data were evaluated following the intention-to-treat and the per-protocol method. Missing data were replaced with baseline data in the intention-to-treat analysis. P values < 0.05 were considered statistically significant. P values between 0.05 and 0.10 were considered borderline significant. Considering a standard deviation of 3.5 kg, the statistical power (α = 0.05; β = 0.80) was sufficient to detect a difference in body weight of 1.3 kg between the two study groups. We used SPSS, version 14.0 (SPSS Inc., Chicago, IL), to perform the statistical analyses.

## Results

### Study population

Characteristics at study entry were comparable between groups, with the exception of sex distribution and fat-free mass (Table 1). Of the 200 participants, 35 subjects were lost during follow-up due to non-compliance with the weight reduction program (n = 23), personal reasons (n = 7), diagnosis of Guillain-Barré syndrome (n = 1), diagnosis of a malignancy requiring chemo preventive therapy (n = 1), and pregnancy (n = 3). The number of drop-outs did not differ between study groups (P > 0.05) (Figure 1). Medication use was similar between groups at baseline (Table 1) and did not change during the study period (data not shown).

**Table 1:** Characteristics of the study groups

Characteristics^1^	DGE group (n = 100)^2^	LOGI group (n = 100)^2^	P value^3^
Men (n)	24	38	0.032
Age (y)	47 (10.8)	47 (10.3)	0.958
Current smoker (n)	43	44	0.887
Height (m)	1.7 (0.1)	1.7 (0.1)	0.153
Weight (kg)	98.8 (16.9)	100.3 (15.9)	0.526
Body Mass Index (kg/m^2^)	33.8 (4.8)	33.5 (3.9)	0.665
Fat mass (kg)	40.9 (11.6)	39.3(10.1)	0.314
Fat-free Mass (kg)	57.5 (10.4)	61.0 (12.2)	0.032
Waist circumference (cm)	108.1 (13.1)	109.9 (10.7)	0.277
Heart rate (beats/min)	70.5 (12.1)	69.5 (11.9)	0.537
Systolic blood pressure (mmHg)	128 (14)	126 (13)	0.408
Diastolic blood pressure (mmHg)	86 (8)	86 (8)	0.858
Triglycerides (mmol/l)	1.39 (0.65)	1.31 (0.56)	0.357
Total cholesterol (mmol/l)	5.54 (1.10)	5.50 (0.93)	0.805
LDL-cholesterol (mmol/l)	3.56 (0.91)	3.54 (0.80)	0.635
HDL-cholesterol (mmol/l)	1.46 (0.37)	1.49 (0.37)	0.612
Glucose (mmol/l)	5.62 (0.85)	5.68 (1.09)	0.650
Fructosamine (μmol/l)	284.2 (28.2)	286.1 (26.4)	0.633
HbA1c (%)	5.6 (0.5)	5.6 (0.5)	0.764
Proinsulin (pmol/L)	9.4 (8.9)	11.5 (14.4)	0.217
Adiponectin (μg/ml)	6.7 (3,7)	6.4 (3.5)	0.642
Metabolic syndrome (%)	54.0	45.0	0.203
Drug therapy (%)			
- Antihypertensive drugs	31.0	24.0	0.268
- Lipid-lowering drugs	4.0	6.0	0.516
- Antidiabetic drugs	1.0	4.0	0.174

^1^mean (standard deviation) and percentage, respectively; ^2^DGE group; high carbohydrate diet; LOGI group; low carbohydrate diet; ^3^unpaired t-test, or Chi-square test when appropriate.

### Energy and macronutrient intake and energy expenditure (intention-to-treat analysis)

Energy and macronutrient intake and energy expenditure are shown in Table 2. At baseline, energy intake, energy expenditure and macronutrient composition were similar in both groups. During the first six months energy intake decreased by approximately 400 kcal/d compared to baseline in both groups. At month 12, mean energy intake had slightly increased again in both groups but remained below baseline values. Metabolic rate increased in both groups. Mean macronutrient composition differed significantly between both groups from month 1 to month 12. During the study period, carbohydrate intake was between 11% and 7% higher in the DGE group compared with the LOGI group. In addition, the LOGI group had a 4% to 8% higher fat intake and a 2% to 3% higher protein intake than the DGE group, whereas alcohol intake did not differ between groups (data not shown). Especially the DGE group did not achieve the target for carbohydrate intake (> 55%).

**Table 2:** Energy and macronutrient intake and energy expenditure (intention-to-treat analysis)

Variable^1^	DGE group (n = 100)^2^	LOGI group (n = 100)^2^	P value^3^
Calories (kcal/d)			
Baseline	2192 (668)	2140 (696)	0.617
1 month	1759 (468)^4^	1697 (503)^4^	0.370
3 months	1755 (478)^4^	1700 (591)^4^	0.474
6 months	1783 (597)^4^	1742 (624)^4^	0.636
12 months	1854 (624)^4^	1866 (710)^4^	0.903
Carbohydrates (% energy)			
Baseline	47.1 (7.9)	44.8 (8.6)	0.065
1 month	49.0 (8.2)	37.7 (11.4)^4^	< 0.001
3 months	49.8 (7.6)^4^	39.7 (11.0)^4^	< 0.001
6 months	49.5 (7.6)^4^	40.9 (10.1)^4^	< 0.001
12 months	50.1 (8.2)^4^	43.5 (9.9)	< 0.001
Fat (% energy)			
Baseline	33.7 (6.9)	35.2 (8.1)	0.169
1 month	30.6 (7.2)^4^	38.1 (9.2)^4^	< 0.001
3 months	29.2 (6.5)^4^	37.1 (9.0)	< 0.001
6 months	29.7 (6.5)^4^	36.5 (9.5)	< 0.001
12 months	30.2 (7.0)^4^	34.2 (8.7)	0.001
Protein (% energy)			
Baseline	16.0 (3.9)	16.8 (3.6)	0.164
1 month	17.8 (4.2)^4^	21.2 (6.1)^4^	< 0.001
3 months	17.8 (4.0)^4^	20.0 (4.3)^4^	< 0.001
6 months	17.7 (4.0)^4^	19.3 (4.7)^4^	0.012
12 months	16.7 (3.1)	18.9 (4.4)^4^	< 0.001
Energy expenditure (kcal/kg/d)			
Baseline	32 (5)	31 (6)	0.864
1 month	33 (6)^4^	33 (7)^4^	0.876
3 months	34 (6)^4^	33 (6)^4^	0.252
6 months	34 (6)^4^	33 (6)^4^	0.315
12 months	34 (5)^4^	33 (6)^4^	0.618

^1^mean (standard deviation) and percentage, respectively; ^2^DGE group; high carbohydrate diet; LOGI group; low carbohydrate diet; ^3^changes between study groups (unpaired t-test); ^4^change from baseline is significantly different within a subgroup (paired t-test).

### Primary endpoints (intention-to-treat analysis)

**Figure 2:**
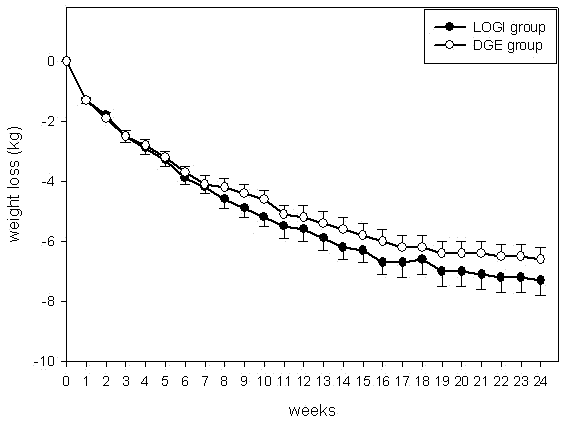
**Weight loss according to weekly telemetric transfer of body weight data during the first 6 study months; results are presented as mean ± standard error of the mean**.

**Figure 3:**
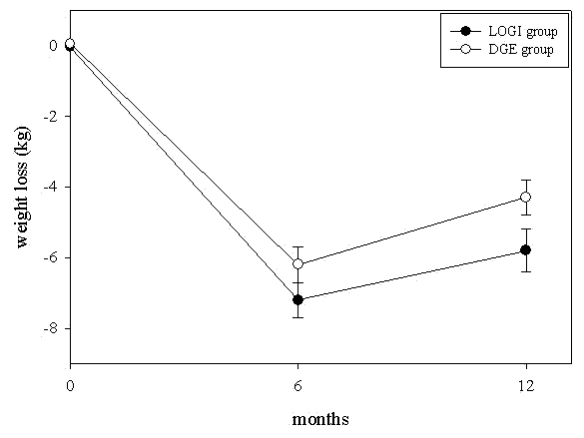
**Weight loss in patients on an energy-restricted high-carbohydrate diet (DGE group) or an energy-restricted low-carbohydrate diet (LOGI group) at baseline and after 6 and 12 months; results are presented as mean ± standard error of the mean**. P = 0.198 between groups at month 6, and P = 0.065 between groups at month 12.

Both diets resulted in similar weight loss (Figures 2 and 3). In detail, weight loss was 7.2 ± 5.4 kg in the LOGI group and 6.2 ± 4.8 kg in the DGE group at month 6. In the second half-year, mean weight regain was 1.6 kg in the LOGI group and 1.9 kg in DGE group. Thus, at the end of the study weight loss was 5.8 ± 6.1 kg in the LOGI group and 4.3 ± 5.1 kg in the DGE group at month 12, a difference that reached borderline significance (p = 0.065). In both groups, approximately 76% of weight reduction was due to a loss of fat mass.

### Secondary endpoints (intention-to-treat analysis)

Waist circumference decreased in both study groups (Table 3). At month 6, values did not differ between groups. At month 12, however, the decrease in waist circumference was more pronounced in the LOGI group compared with the DGE group even when adjustments were made for fat-free mass, and sex distribution. Triglyceride levels did not change from baseline to month 6 and month 12 in the DGE group, but declined in the LOGI group by 14% at month 6, and 7% at month 12 compared to baseline values. HDL-cholesterol decreased in the DGE group until month 6, but rose again in the second half-year, whereas this parameter remained unchanged in the LOGI group. Changes from baseline in triglyceride and HDL-cholesterol levels differed significantly between the LOGI and DGE groups at month 6. However, these differences disappeared later on in the study. Total-cholesterol was not affected in the LOGI-group but increased in the DGE group between month 6 and 12. Nevertheless, total cholesterol did not differ between groups at any time point. LDL-cholesterol did not change during the entire study period, neither in the DGE group nor in the LOGI group. Diastolic blood pressure decreased in both groups, whereas systolic blood pressure was significantly higher at month 12 in the DGE group compared with the LOGI group. The differences in systolic blood pressure at month 12 remained significant after adjustments were made for fat free mass and sex distribution. Changes in parameters of glucose metabolism were similar between groups (Table 3). Results did not differ when the per protocol method was used (data not shown).

**Table 3:** Changes in body composition, metabolic and cardiovascular risk factors (intention-to-treat analysis)

Variable^1^	DGE group (n = 100)^2^	LOGI group (n = 100)^2^	P value^3^
Fat mass (kg)			
6 months	-4.6 (3.7)^4^	-5.6 (4.2)^4^	0.073
12 months	-3.3 (4.4)^4^	-4.2 (4.9)^4^	0.132
Fat free mass (kg)			
6 months	-1.6 (2.1)^4^	-1.6 (2.5)^4^	0.740
12 months	-1.3 (3.6)^4^	-1.5 (2.5)^4^	0.794
BMI (kg/m^2^)			
6 months	-2.1 (1.6)^4^	-2.3 (1.8)^4^	0.250
12 months	-1.5 (1.8)^4^	-1.9 (2.1)^4^	0.110
Waist circumference (cm)			
6 months	-6.6 (5.3)^4^	-8.0 (5.5)^4^	0.083
12 months	-4.7 (8.9)^4^	-6.9 (6.1)^4^	0.037
Systolic blood pressure (mmHg)			
6 months	-4 (15)^4^	-6 (16)^4^	0.102
12 months	-1 (15)	-5 (14)^4^	0.007
Diastolic blood pressure (mmHg)			
6 months	-3 (9)^4^	-3 (8)^4^	0.884
12 months	-2 (8)^4^	-3 (9)^4^	0.440
Triglyceride (mmol/l)			
6 months	-0.03 (0.55)	-0.18 (0.40)^4^	0.005
12 months	-0.04 (0.50)	-0.10 (0.47)^4^	0.164
Total cholesterol (mmol/l)			
6 months	-0.07 (0.50)	-0.07 (0.56)	0.926
12 months	0.13 (0.61)^4^	0.03 (0.75)	0.259
LDL-cholesterol (mmol/l)			
6 months	-0.03 (0.51)	-0.03 (0.50)	0.921
12 months	0.06 (0.59)	0.02 (0.65)	0.564
HDL-cholesterol (mmol/l)			
6 months	-0.09 (0.19)^4^	-0.02 (0.20)	0.005
12 months	-0.03 (0.17)	-0.02 (0.21)	0.668
Glucose (mmol/l)			
6 months	-0.28 (0.59)^4^	-0.26 (0.76)^4^	0.475
12 months	-0.14 (0.46)^4^	-0.25 (0.75)^4^	0.235
HbA1c (%)			
6 months	-0.2 (0.2)^4^	-0.2 (0.2)^4^	0.840
12 months	-0.2 (0.2)^4^	-0.2 (0.2)^4^	0.314
Fructosamine (μmol/L)			
6 months	-0.4 (27.5)	1.1 (24.6)	0.960
12 months	-5.9 (24.6)^4^	-8.8 (31.1)^4^	0.580
Proinsulin (pmol/L)			
6 months	-1.4 (11.4)	-3.7 (14.8)	0.676
12 months	-2.2 (8.2)^4^	-5.0 (14.0)^4^	0.148
Adiponectin (μg/ml)			
6 months	-0.2 (3.4)	-0.1 (3.0)	0.929
12 months	0.9 (2.7)^4^	1.3 (2.6)^4^	0.288

^1^mean (standard deviation) and percentage, respectively; ^2^DGE group; high carbohydrate diet; LOGI group; low carbohydrate diet; ^3^analysis of covariance with baseline values as covariates. ^4^change from baseline is significantly different within a subgroup (paired t-test).

## Discussion

In this telemedically guided weight reduction program both, an energy-restricted high-carbohydrate diet (DGE group) and an energy-restricted low-carbohydrate diet (LOGI group) resulted in a satisfactory weight loss and an improvement of several metabolic parameters over a period of 12 months. However, weight loss tended to be greater, and the decline in waist circumference and systolic blood pressure was more pronounced in the LOGI group than in the DGE group. With respect to HDL-cholesterol and triglycerides, beneficial effects in the LOGI group compared to the DGE group were only seen at the 6-month follow-up visit. Since mean energy intake and energy expenditure did not differ between the two groups, the differences in the aforementioned parameters are most likely due to differences in macronutrient relations.

The mean body weight loss of 7.2 kg at month 6 and 5.8 kg at month 12 in the LOGI group (Figure 3) was similar compared to literature reports about efficacy of carbohydrate-restricted diets [12-15,29]. In these earlier reports, results of carbohydrate-restricted diets were also compared with fat-restricted diets. Mean weight loss of the fat-restricted diets was only 1.6 kg – 3.9 kg at month 6 [12-15,29] and 2.2 kg – 3.1 kg at month 12 [12,14,29] and thus lower than the mean weight loss in the energy-restricted fat-reduced group (DGE group) in our study (6.2 kg at month 6 and 4.3 kg at month 12, respectively). It has been assumed that the better 1-year efficacy of carbohydrate-restricted diets on weight loss compared to fat-restricted diets can be explained by a greater energy deficit in subjects on a low-carbohydrate intake. Since in our study energy intake and body weight loss were similar in both groups within the first 6 month, our results are in line with the aforementioned hypothesis. However, despite similar energy intake at month 12, weight loss tended to be greater at month 12 in the LOGI group compared with the DGE group of our study. Note that differences in macronutrient composition of the diet may influence resting or postprandial energy expenditure [15]. Since protein intake increases diet-induced thermogenesis, it can well be that the slightly higher protein intake in the LOGI group compared with the DGE group during the entire study period could also have resulted in a slightly higher metabolic rate, and thus in a slightly higher weight loss. Nevertheless, our data also demonstrate that even with an energy-restricted high-carbohydrate diet a weight loss similar to an energy-restricted carbohydrate-reduced diet can be achieved. Importantly, the regain of weight after 12 months confirms earlier assumptions [17,18] that continuous intensive care and control is a more important factor for participants' compliance and, thus, for the success of a weight loss program than minor alterations in macronutrient composition in the diet. Our results are in line with a very recently published trial by Sacks et al. [30]. They investigated four diets with different macronutrient relations and showed that weight loss is related to adherence and attendance to instructional sessions. All diets were equally successful in promoting weight loss and maintaining weight.

Despite the favourable effects of both diets on weight loss in our study, the carbohydrate-reduced diet was more beneficial with respect to some cardiovascular risk markers such as waist circumference, triglycerides, HDL-cholesterol, and systolic blood pressure compared to the fat-reduced diet. Our data show into the same direction than earlier study results [12-14,29,31,32] but were less pronounced. This may at least in part be due to two factors: the comparably higher weight loss by carbohydrate-restricted diets in these earlier studies [12,13,29,30], and/or a more pronounced carbohydrate restriction in the low-carbohydrate diets used [12-14,29,31].

Low-carbohydrate diets may diminish triglyceride production in the liver in response to decreased carbohydrate delivery [14]. The greater preservation of HDL-cholesterol on a low-carbohydrate diet may be the result of down-regulation by dietary fats of those hepatic receptors, which bind HDL-cholesterol [33]. However, it remains unclear why HDL-cholesterol and triglycerides differed only at the 6-month visit between the two groups of our study. It may well be that the transient effects on triglycerides and HDL-cholesterol are related to differences in macronutrient composition in our study groups which became smaller over time (Table 3). Obviously, other variables like waist circumference and systolic blood pressure were only influenced by the degree of weight loss in the two groups and not by macronutrient composition (Table 3).

Our results also demonstrate the general problem of adherence to a diet with extreme nutrition relations. The use of telemedicine permits continuous contact to participants, individual support, and control of weight loss. Moreover, use of this technique resulted in a low drop-out rate of only 17% in our study participants. Nevertheless, it was not possible to achieve the target macronutrient relations in the two study groups in the long run. The target of carbohydrate content in the LOGI group (< 40% energy) was only reached within the first 3 months and the target of carbohydrate content in the DGE group (>55% energy) was not reached at any time. Note that earlier investigations have already reported relatively high attrition-rates of 30–50% in studies using very low-carbohydrate diets [12,34]. Despite some additional beneficial effects of energy-restricted low-carbohydrate diets on cardiovascular risk markers it appears that poor long-term adherence to such diets limits its success in clinical practise [34].

It should also be mentioned that the similar loss in body weight in both groups of our study was associated with a similar improvement in several metabolic risk markers such as fat mass, diastolic blood pressure, and glucose, fructosamine, proinsulin, and adiponectin blood concentrations (Table 3). Decreasing insulin resistance and increasing adiponectin levels reduces atherosclerotic and inflammatory processes and endothelial dysfunction [35-37] and may thus have decreased the cardiovascular risk in both study groups.

## Conclusion

Despite the favourable effects of both diets on weight loss, the carbohydrate-reduced diet was more beneficial with respect to major cardiovascular risk factors such as central obesity, triglycerides, HDL-cholesterol, and systolic blood pressure compared to the fat-reduced diet. Nevertheless, compliance with a weight loss program appears to be even a more important factor for success in prevention and treatment of obesity than the composition of the diet.

## Competing interests

The authors declare that they have no competing interests.

## Authors' contributions

The authors responsibilities were as follows – SF: study design, recruitment of patients, data collection, statistical analysis, data interpretation, and drafting the manuscript; AZ: study design, statistical analysis, data interpretation, and drafting the manuscript; HKB and HK: study design, and critical revision of the manuscript for important intellectual content; CG, JK and KK: data analysis and critical revision of the manuscript for important intellectual content; PS: data interpretation, and critical revision of the manuscript for important intellectual content. All authors approved the final manuscript.
